# Surgical outcomes of primary carcinosarcoma of the gallbladder after curative resection: A rare case series

**DOI:** 10.1016/j.ijscr.2019.10.056

**Published:** 2019-10-29

**Authors:** Mohammed Yousef Aldossary, Abdullah Saleh AlQattan, Yasmeen Megad Alghamdi, Amal A. Alayed, Fatimah Alquraish, Omar Abdulaziz AlAnzi, Nawaf Alabdulrahim, Abdulaziz Alateeq, Mohammed Saad Alqahtani

**Affiliations:** a Department of General Surgery, Hepatobiliary Surgery Unit, King Fahad Specialist Hospital-Dammam, Saudi Arabia; b Department of General Surgery, Northern Area Armed Forces Hospital, Saudi Arabia; c Department of General Surgery, King Abdulaziz Airbase Armed Forces Hospital, Dhahran, Saudi Arabia; d College of Medicine, King Faisal University, Al-Ahsa, Saudi Arabia

**Keywords:** CEA, carcinoembryonic antigen, CA19.9, carbohydrate antigen 19.9, AFP, α-fetoprotein, CT, computed tomography, CK, cytokeratin, AJCC, American Joint Committee on cancer, PET, positron emission tomography, Carcinosarcoma, Gallbladder, Prognosis, Surgical outcome, Survival rate

## Abstract

**Introduction:**

Primary carcinosarcoma is a rare neoplasm of the gallbladder, and is characterized by collision elements of the adenocarcinomatous and sarcomatous components. The tumours comprise both malignant epithelial and mesenchymal elements, and are usually very aggressive, conferring poor prognosis. Adenocarcinomas are the most common neoplasms of the gall bladder, while carcinosarcoma are extremely rare, with a global prevalence rate of less than 1%. The low incidence of carcinosarcoma greatly hinders the understanding of its aetiology.

**Presentation of Case:**

Here, we report the surgical outcome of 3 rare cases of gallbladder carcinosarcomas, two of which had a IVB stage disease and in one case the staging was II. Following surgery, the survival in the former was less than 1 year, while in the latter, it extended up to 7 years (86 months), and the patient is alive till date. This study aimed to elucidate the surgical outcomes and prognosis after curable resection for carcinosarcomas of the gallbladder.

**Discussion:**

The findings suggest that carcinosarcomas are associated with a poor prognosis and short survival, particularly in those with tumours of >5 cm. In the absence of effective adjuvant treatment, surgery remains the mainstay of treatment.

**Conclusion:**

Complete surgical resection may improve prognosis and extend survival in patients with this rare and aggressive form of gallbladder cancer.

## Highlights

• Primary carcinosarcoma is a rare neoplasm of the gallbladder.
• It is characterised by collision of adenocarcinomatous and sarcomatous components.
• This series shows that complete resection is the only means of extending survival.
• The role of adjuvant therapy remains unclear.

## 1 Introduction

Carcinosarcoma is also known as sarcomatoid carcinoma, spindle cell carcinoma, and pseudosarcoma [1]. Gallbladder carcinomas are rare in the Middle Eastern and Western regions of the world [2]; however, they are one of the most common neoplasms of the hepato-biliary system in north India [3]. The most common histological type is adenocarcinoma (80–95%); the other histological types such as anaplastic/undifferentiated (2 to 7%), squamous cell (1 to 6%), adenosquamous (1 to 4%), and small cell (1 to 3%) carcinomas are rare, while carcinosarcomas are extremely rare, with a prevalence rate of less than 1% worldwide [[4], [5], [6]]. Carcinosarcomas are characterized by the collision of adenocarcinomatous and sarcomatous components [7]. The tumours comprise both, epithelial and mesenchymal elements [8]. These components comprise adenocarcinoma and undifferentiated spindle or satellite cells, respectively [9]. Diverse heterogeneous elements such as those of chondrosarcoma, osteosarcoma, rhabdomyosarcoma, and leiomyosarcoma often accompany the mesenchymal components [10], that may proliferate in an intermixed or disconnected fashion [11]. Carcinosarcoma is commonly encountered in many other different organs, including the kidneys, pancreas, oesophagus, lungs, and uterus [[12], [13], [14], [15]]; however, it is extremely rare in the gallbladder [5]. Dismal survival rates ranging from 2.9 to 6 months have been reported in patients despite curative resection [16]. This study aimed to elucidate the surgical outcomes and prognosis of patients who underwent curative resections for carcinosarcomas of the gallbladder. This case series has been reported according to surgical case series criteria [17].

## 2 Presentation of case

### 2.1 Case 1

**Fig. 1:**
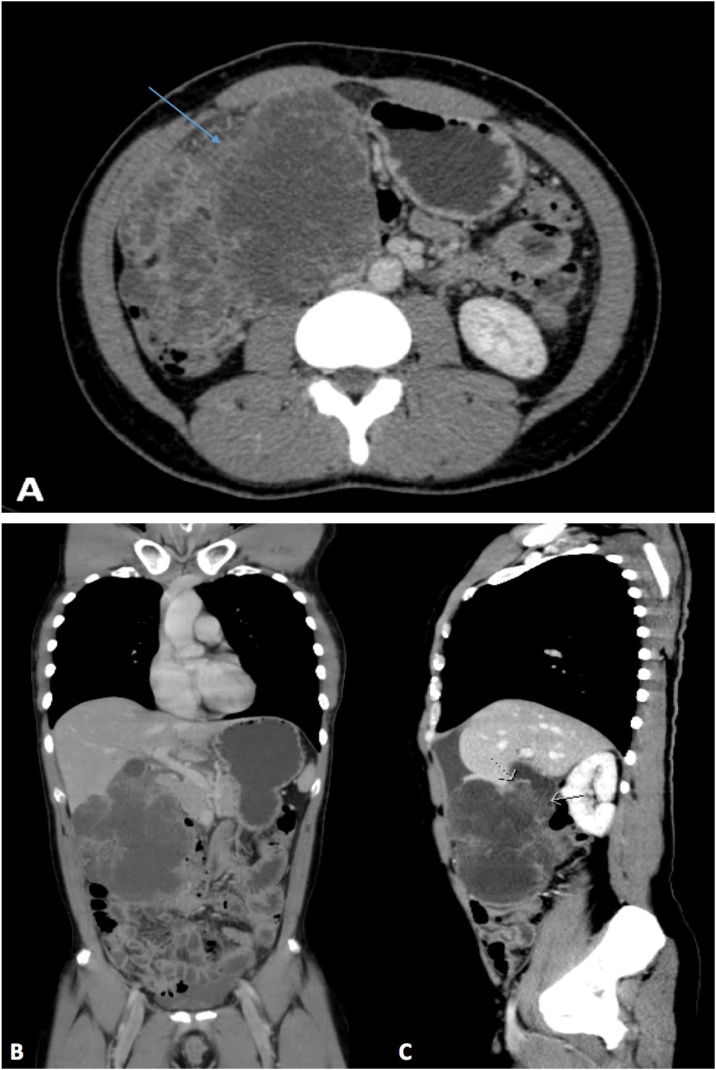
A: Computed tomography scans of the chest and the abdomen revealing a large polypoidal enhancing mass in the gallbladder measuring 11.5 × 9.2 × 5 cm with focal lesions in segment V. B: Coronal view of computed tomography scans of the chest and abdomen revealing a large gallbladder mass. C: Sagittal view of the same image.

A 40-year-old male, known to have sickle cell disease with a history of several admissions for sickle cell crisis, was referred to our surgical outpatient department with complains of severe intermittent pain in the right hypochondrium for 7 months. He ignored the pain, considering it to be related to the chronic pain of sickle cell disease. He denied any history of anorexia, weight loss, jaundice, or gallstones. General physical examination revealed a thin body build without pallor or jaundice; a palpable mass with tenderness in the right hypochondrium was found on abdominal examination. Laboratory examination demonstrated the following: haemoglobin: 8.5 g/dl, leucocyte count: 6.2 × 10^9^/L, haematocrit: 28.2%, and platelet count: 672 × 10^9^/L. The C-reactive protein level was 174.1 mg/L. Liver function tests demonstrated the following: albumin, 23 g/L, total protein: 65 g/L, alanine aminotransferase: 21 units/L, aspartate transaminase: 27 units/L, alkaline phosphatase: 52 units/L, total bilirubin: 11.32 umol/L, conjugated bilirubin: 6.28 umol/L, and amylase: 22 units/L. The coagulation profile was within the normal range. Cancer antigen markers including carcinoembryonic antigen (CEA), carbohydrate antigen 19.9 (CA19.9), and α-fetoprotein (AFP) were all within the normal range. Initial ultrasound of the abdomen revealed a large mass encasing the gallbladder, with multiple small gallstones. Computed tomography (CT) of the chest, abdomen, and pelvis revealed a large polypoidal enhancing mass in the gallbladder measuring 11.5 × 9.2 × 5 cm, and focal lesions in segment V of the liver with extension to the right colon; peritoneal deposits were also observed (Fig. 1A, B, and C). Open laparotomy revealed a huge mass originating from the liver and gallbladder, which was adherent to the transverse colon, and was occupying most of the right side of the abdomen. Multiple small peritoneal nodules were also found; samples were obtained and sent for frozen section. However, the results were inconclusive. An extended right hemicolectomy was performed with ileocolic anastomosis to restore gastro-intestinal continuity; radical cholecystectomy with excision of the tumour, and resection of segment V were also performed.

**Fig. 2:**
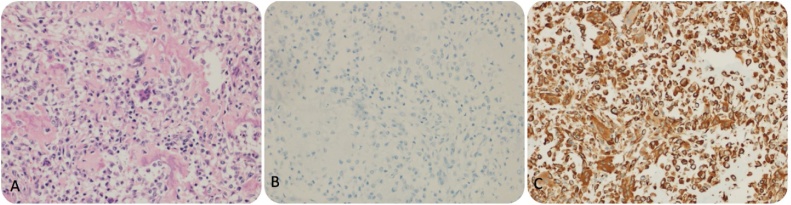
A: HE staining (20×) of the epithelial component of carcinosarcoma. B: Negative on CK staining. C: Vimentin positivity in the sarcomatous component.

The surgical specimen included a huge friable mass of variegated tissue measuring 13 cm in the greatest dimension. Histopathological examination revealed a malignant tumour cells included epithelial components of carcinosarcoma, and were positive for vimentin and negative for cytokeratin (CK) (Fig. 2A, B, and C). The tumour demonstrated infiltrative margins, involving the gallbladder, colon, liver, and omentum. The liver and transverse colon were involved by the tumour, and the cystic duct was free.

The patient was diagnosed with metastatic gallbladder carcinosarcoma of stage IVB (pT3, pN0, M1) based on the 7th edition of the American Joint Committee on cancer (AJCC) staging system. He was then referred to the medical oncology department, where he received 2 cycles of adjuvant chemotherapy. Hepatic recurrence was observed with multiple variably sized nodules in the omentum 3 months after surgery, and he died 3 months later.

### 2.2 Case 2

**Fig. 3:**
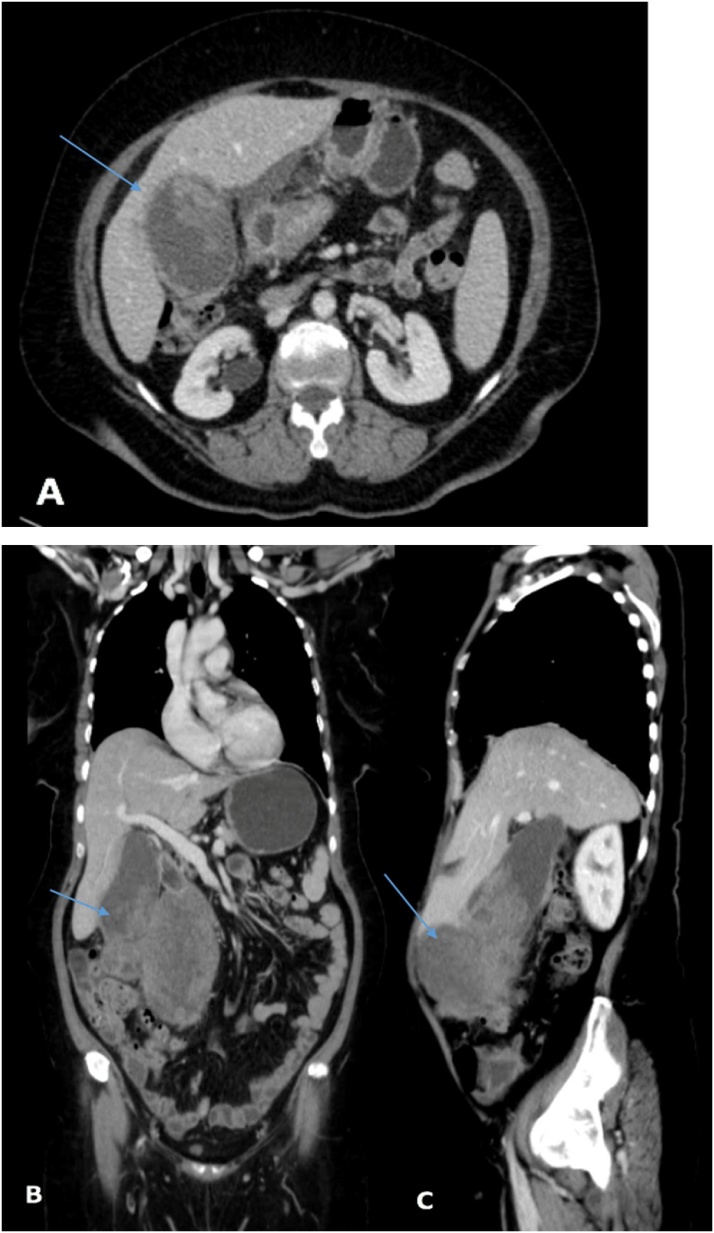
A: Computed tomography scans of the chest and the abdomen revealing a distended gallbladder fundus with intraluminal tumour and huge exophytic mass measuring approximately 13.6 × 12 × 9.5 cm. B: Coronal view of the computed tomography scans of the chest and abdomen revealing a large gallbladder mass; medially the lesion is displacing the distal stomach at the level of pylorus, and the mass protruding downward is displacing the transverse colon inferiorly. Few small portal lymph nodes are observed. C: Sagittal view of the same image.

A 52-year-old female with no known history of any medical illness presented to the emergency department of the referring hospital with complains of right upper quadrant pain for the past 5 months. The pain was intermittent, increased with fatty meals, and was radiating to the back; it was associated with multiple episodes of vomiting (clear liquid) in the past 5 days prior to presentation. The patient denied any history of anorexia, weight loss, jaundice, or gallstones. On general physical examination, the patient was well-nourished, in moderate pain, and had no pallor or jaundice. Abdominal examination revealed minimal tenderness in the epigastric area with a palpable mass in the right upper quadrant, 7 cm below the costal margin. Laboratory examination revealed the following: haemoglobin: 10.7 g/dl, leucocyte count: 9.15 × 10^9^/L, haematocrit: 32.5%, and platelet count: 423 × 10^9^/L. Liver function tests showed the following: albumin: 34 g/L, total protein: 66 g/L, alanine aminotransferase: 55 units/L, aspartate transaminase: 52 units/L, alkaline phosphatase: 115 units/L, total bilirubin: 6.9 umol/L, conjugated bilirubin: 23.3 umol/L, and amylase: 39 units/L. The coagulation profile was within the normal range, and the cancer antigen markers revealed a CA19.9 level of 154.3 IU/mL, with normal levels of AFP and CEA. Initial ultrasound of the abdomen, performed in the referring hospital demonstrated thickening of the gallbladder wall, which measured approximately 17 mm, with large irregular soft tissue masses arising from the fundus, that measured 8 × 6 cm. These were seen infiltrating the surrounding sub-hepatic fat planes, and were inseparable from the transverse colon; no dilated intrahepatic biliary radicals, focal hepatic lesions, or enlarged lymph nodes were noted. CT of the chest, abdomen, and pelvis revealed distension of the gallbladder fundus with an intraluminal tumour and huge exophytic mass measuring approximately 13.6 × 12 × 9.5 cm; medially, the lesion displaced the distal stomach at the level of pylorus, and the mass protruding downwards displaced the transverse colon inferiorly. Few small portal lymph nodes were also seen (Fig. 3A, B, and C). Open laparotomy revealed a mass adherent to the transverse colon, greater omentum, and transverse meso-colon, and the tumour was free from the surrounding structures. The patient underwent radical cholecystectomy, transverse colectomy, and distal gastrectomy with Roux-en-Y gastrojejunostomy; colonic continuity was achieved using side-to-side transverse anastomosis. Falciform ligament resection with limited lymphadenectomy was performed to remove the peri-pancreatic lymph nodes. The surgical specimen included a huge friable variegated tissue mass, measuring 13 cm in the greatest dimension.

**Fig. 4:**
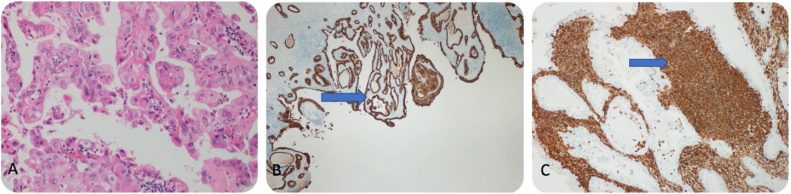
A: HE staining (20×) of the epithelial component of carcinosarcoma. B: CK staining positivity in the carcinoma component. C: Vimentin positivity in the sarcomatous component.

Histopathological examination revealed a huge polypoidal tumour measuring 11.0 cm, filling the lumen of the gallbladder; the findings were consistent with those of carcinosarcoma, revealing an anaplastic sarcomatous component with a heterologous chondrosarcomatous components. The large omental mass measuring 20 × 10 cm showed an anaplastic poorly differentiated tumour consistent with metastatic carcinosarcoma; the resected liver showed direct invasion by carcinosarcoma (with dominance of the sarcomatous component) from the gallbladder. No direct invasion of the muscularis propria, submucosa, or mucosa was observed. However, peri-colonic fatty tissue invasion was seen. The stomach was not directly involved, there was only an invasion of the peri-gastric fatty tissue. The greater omentum and falciform ligament showed no metastases; 4 of 8 peri-pancreatic lymph nodes showed metastatic poorly differentiated carcinosarcoma. Immunohistochemical staining revealed positivity for both, CK (carcinomatous component) and vimentin (sarcomatous component) stains (Fig. 4A, B, and C).

Therefore, the patient was diagnosed with metastatic carcinosarcoma of the gallbladder of stage IVB (pT4, pN2, M1). The patient was referred to the medical oncology department; however, she refused adjuvant chemotherapy, and presented with recurrence involving multiple segments of the liver 2 months after surgery. She died 1 month later.

### 2.3 Case 3

**Fig. 5:**
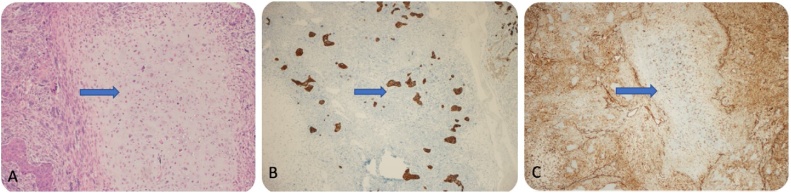
A: HE staining (20×) of the epithelial component of carcinosarcoma. B: CK staining positivity in the carcinomatous component: C: Vimentin positivity in the sarcomatous component.

A 62-year-old female, with a history of hypertension and diabetes mellitus underwent laparoscopic cholecystectomy after being referred to our hospital; histopathological examination revealed carcinosarcoma of the gallbladder. The patient had presented to the emergency department of the referring hospital with complains of severe intermittent right upper quadrant pain for the past two months, with nausea and anorexia. The pain increased with fatty meals and did not radiate; she denied any history of vomiting, weight loss, jaundice, and gallstones. On general physical examination, she was well-nourished, in moderate pain, and was not pale or jaundiced. Abdominal examination revealed moderate tenderness in the right upper quadrant. Laboratory examination revealed: haemoglobin level: 2.7 g/dl, leucocyte count 6.8 × 10^9^/L, haematocrit: 40.0%, and platelet count: 325 × 10^9^/L. The liver function test showed: albumin: 39 g/L, total protein: 76 g/L, alanine aminotransferase: 39 units/L, aspartate transaminase: 17 units/L, alkaline phosphatase: 67 units/L, total bilirubin: 3.85 umol/L, and amylase: 49 units/L. The coagulation profile was within the normal range, and cancer antigen markers CEA, CA19.9, and AFP were all within normal ranges. Ultrasound of the abdomen, performed in the referring hospital showed a septate gallbladder with large stones measuring 1.6 × 1.2 cm, and another non-mobile echogenic structure measuring 2.7 × 0.9 cm in the dependent part, suggestive of a focal wall thickening or polyp. She underwent elective laparoscopic cholecystectomy in the referring hospital. The intraoperative findings revealed a distended gallbladder with an hour-glass appearance, suggestive of empyema; focal wall thickening was observed in the body of the gallbladder with an intraluminal mass measuring 2 × 2 cm. Histopathological examination revealed an intraluminal tumour measuring 2 × 2 cm, that appeared to be a well to moderately differentiated carcinosarcoma of the gallbladder. It had invaded the peri-muscular connective tissue through the muscularis layer; no lymph nodes were identified. Immunohistochemical stains showed positivity for both, CK and vimentin stains in the carcinomatous and sarcomatous components, respectively (Fig. 5A, B, and C). The tumour was of stage II (pT2, pN0, Mx). The patient was referred to our hospital for further investigations and management following the diagnosis of carcinosarcoma. CT revealed a post cholecystectomy status with no suggestion of residual tumour or liver invasion, and no evidence of distension or metastasis. A positron emission tomography (PET) scan revealed no hypermetabolic lesions. She underwent open completion radical cholecystectomy, liver bed resection with hepatic lymphadenectomy, segment IV and V resection, and port site excision. The falciform ligament was excised along with the hepatic artery lymph nodes, and were sent for histopathological examination; the findings demonstrated all port sites, hepatic artery lymph nodes, falciform ligament, and liver segments IV and V to be negative for malignancy.

**Fig. 6:**
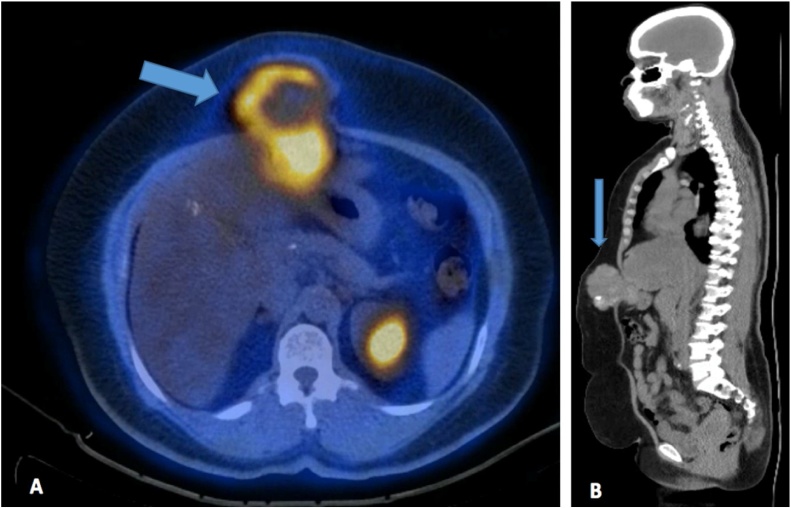
A: Positron emission tomography-CT of chest, abdomen, and pelvis revealing post-surgical changes in the liver with no focal uptake suggestive of local recurrence. Fatty changes are noted. A soft tissue lesion with areas of coarse calcification is seen in the anterior abdominal wall on the right, next to the midline; it is in close proximity to the site of the previous surgical incision, and measures 8.3 × 6.9 cm, with a maximum standardized uptake value of 6.5. B: Sagittal view of the same image.

**Fig. 7:**
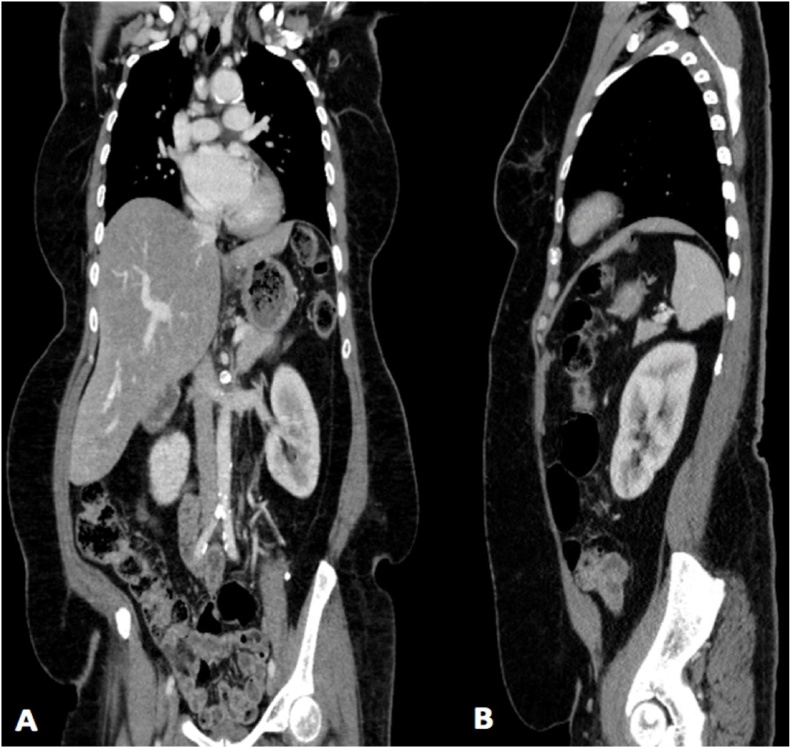
A: Coronal view of computed tomography scans of the chest and abdomen after 7 years from first surgery showing no local recurrence and no new lesions. B: Sagittal view of the same image.

She was therefore diagnosed with incidental gallbladder carcinosarcoma of stage II (pT2, pN0, M0). The patient was referred to the medical oncology department where she received 14 cycles of adjuvant chemotherapy, and was on regular follow up. After 2 years of regular follow up, the CT revealed a localised anterior wall collection with small peri-hepatic fatty densities. The PET-CT scans of the chest, abdomen, and pelvis demonstrated a soft tissue lesion with areas of coarse calcification in the anterior abdominal wall, next to the midline measuring 8.3 × 6.9 cm; it was located near the site of the previous surgical incision (Fig. 6A and B). Ultrasound-guided fine needle aspiration of the abdominal soft tissue mass revealed malignant tumour cells, that were positive for vimentin; this confirmed recurrence in this known case of carcinosarcoma. The cancer antigen markers showed elevated levels of CA19.9 (46.54 IU/mL), with normal levels of AFP and CEA. Based on these findings, she was diagnosed with abdominal wall port-site metastasis, and underwent open laparotomy for the second time. The intraoperative findings revealed a mass attached to the liver and stomach wall. Wide local excision of the mass was performed with wedge resection of the liver, lymph nodes, and part of the stomach; plastic reconstruction of the abdominal wall was also performed. Histopathological examination revealed the presence of sarcoma in the abdominal wall, stomach, and liver, with no lymph node metastases. The liver, stomach, and surgical resection margins of the skin tested negative for malignancy. She is on regular follow-up, and visits every six months. Her overall survival till date is 7 years and 2 months (86 months) from the first surgery, with no further recurrence of the tumour (Fig. 7A and B).

## 3 Discussion

In our case series, all 3 patients had tumour recurrence at 3 months, 2 months, and 25 months after the surgery, respectively. The shortest and longest periods of survival were 3 and 86 months. Two patients died from the tumour recurrence and/or the disease progression, while 1 is alive till date.

The tumour size was greater than 5 cm in 2 of 3 patients; these patients had survived for 3 and 6 months. The patient with a tumour size of less than 5 cm is surviving for 86 months (7 years and 2 months) from the first surgery. The prognosis of gallbladder carcinosarcoma was therefore found to be very poor despite complete resection of the tumour. Carcinosarcoma of the gallbladder was first reported by Landsteiner et al. in 1907. The low incidence of this entity is a major hindrance to the understanding of its aetiology. In view of the absence of a definite staging system for this rare variant, most of the reported cases are staged based on the tumour-node-metastasis staging system for gallbladder cancer. The incidence rate of carcinosarcoma is approximately 1.2 per 100,000 population per year [18,19]. Carcinosarcomas of the gallbladder are usually very aggressive and the prognosis is poor, with a median survival of 7.8 months or less [16,20]. In addition, their clinical behaviour is comparatively poorer than that of carcinomas [21]. According to reports, a 75-year-old male patient with carcinosarcoma survived for 30 months following surgical resection and 6 cycles adjuvant chemotherapy with an oxaliplatin-based regimen [22]. Gao et al. [7] analysed 20 studies from the literature, including 21 patients (8 male and 13 female) between 1980 and 2013; 12 patients demonstrated longer survival following surgery, with follow up periods of up to 60 months. A total of 8 patients demonstrated short survival, with periods ranging from 1.5 to 17 months (mean survival: 6.4 months); the survival in 1 patient was unknown. Nishihara et al. [23] compared the prognoses of 224 patients diagnosed with adenocarcinomas of the gallbladder with that of 9 diagnosed with carcinosarcomas; the survival in the latter and former were up to 9 and 81 months, respectively. They concluded that carcinosarcomas confer considerably poorer prognosis compared to adenocarcinomas. The longest survivor, reported in 2002 [24], had survived for 5 years and 7 months post-operatively. In our cohort, the third patient is surviving for 7 years and 2 months (86 months); this is the longest survival reported in the literature till date.

Tumour size may influence the prognosis and survival rate in these patients. In the meta-analysis conducted by Zhang L et al. in 2008 [25], patients with tumours larger and smaller than 5 cm had a median survival of 5 and 11 months, respectively. In the cohort studied by Gao et al. [7], 8 of 21 patients (8 male and 13 female) died after surgical resection; the tumour size was greater and lesser than 5 cm in 7 and 1 cases, respectively (mean survival: 6.4 months). Overall, among the 12 surviving cases, the tumour size was greater and lesser than 5 cm in 9 and 4 patients, respectively; the duration of follow up after surgery extended for up to 60 months, and that of 1 patient was unknown. In our series, 2 of 3 patients had tumour sizes greater than 5 cm, with survival for up to 6 and 8 months, respectively. In the patient with a tumour of less than 5 cm, the survival has extended for up to 86 months (7.2 years) from the first surgery, and she is on regular follow up. The median survival in carcinosarcoma of the gallbladder extends for up to few months despite complete surgical resection [5,26]. The definitive treatment of this entity is surgical resection, which provides the only means of extending survival 26]. There is no evidence to support the therapeutic benefit of adjuvant chemotherapy or radiation therapy in these cases [27,28]. Usually, the tumours recur in the first 6 months after surgery, and the mean time to recurrence is approximately 50 days [27,29,30].

## 4 Conclusion

The present case series of 3 cases of carcinosarcoma of the gallbladder shows that in this extremely rare and aggressive variant of cancer, surgical resection is the only means of obtaining extended survival. The post-operative survival of 86 months noted in 1 patient from our series who had a tumour smaller than 5 cm, is probably the longest reported in the literature. Cumulative evidence from further cases is needed to improve understanding on this rare and aggressive malignancy.

## Declaration of Competing Interest

The authors declare no conflict of interest.

## Sources of funding

This study did not receive any funding from governmental or private organizations.

## Ethical approval

Ethical approval was obtained from the Institutional Review Board of the King Fahad Specialist Hospital, Dammam, Saudi Arabia. Reference number (SUR0333) Dated 26/09/2019.

## Consent

Written informed consent was obtained from all of the patients for publication of this case series and accompanying images. A copy of the written consent is available for review by the Editor-in-Chief of this journal on request.

## Author contribution

Study concept or design – MYD, MSQ.

Data collection – ASQ, AAA, YMG, FQ, AA, OAA.

Data interpretation – MYD, ASQ, FQ, OAA, NA.

Literature review – MYD, ASQ, YMG, AA, NA.

Drafting of the paper – MYD.

Editing of the paper – MYD, ASQ, MSQ.

## Registration of research studies

Researchregistry5150.

## Guarantor

Mohammed Yousef Aldossary.

## Provenance and peer review

Not commissioned, externally peer-reviewed.
